# Malarial Hæmaturia

**Published:** 1885-10

**Authors:** S. Leard Keown

**Affiliations:** Kemp, Texas


### MALARIAL HÆMATURIA.

*By S. Leard Keown, M. D., Kemp, Texas.*

For Daniel's Texas Medical Journal.

THE occurrence of three fatal cases of malarial hæmaturia within a small radius near this place—and I am pained to state that one had for its subject one of our most estimable and useful physicians, Dr. Peacock, of Prairieville—reminds us of the importance of a thorough understanding of the pathology and treatment of this, the most dreaded of malarial diseases.

The effects of the poison, malaria, upon the vaso-motor centers is paralysis, causing relaxation of the blood vessels and allowing exudation of blood through their walls. The mischief done is more conspicuously seen upon the mucous surfaces, because these are vascular parts with the vessels sparingly supported by substantial tissues. No matter whether the kidneys, stomach, bowels or skin be most involved, the indications for treatment are the same, which are to increase arterial tension, overcome relaxation of the vessels, and check the waste of blood, which is not only being rapidly lost, but its presence everywhere outside of the vessels, seriously interferes with the performance of the functions of organs, and almost totally suspends nutrition and elimination.

Prof. Henry Orendorf, professor of therapeutics in the Kentucky School of Medicine, taught in his lectures and clinics the use of strychnia in this disorder. The same author states, in the Louisville Medical News of August 26, 1882: "This drug (strychnia), standing at the head of vaso-motor stimulants, is especially useful in low vascular tension. It should be administered in full doses, that the relaxed vessels may be made so tense as to prevent exudation. By full doses is meant the amount required to produce the desired effect i. e., to stop leakage. Theaefore, if one thirtieth of a grain does not suffice, push it to one-twentieth, to one-fifteenth, and even to one-tenth of a grain, repeating sufficiently often to secure and keep secured the physiological action of the drug."

The pathological condition being so plain, and the mode of action of strychnia so clear, that my confidence was readily given to the plan of treatment which has been verified in three cases. The symptoms in these cases showed well marked appearances of the malady, with vomiting of "coffee-ground" material, and the passage of blood in the urine and feces. To allay vomiting, and check the discharges from the bowels, opium was given. Calomel in moderate doses was used to assist the eliminative organs. Strychnia was freely used hypodermically, as much of the time as could be alloted to the patient, watching its effects closely and pushing its use to a point barely within the range of its tonic effect. During my absence fld. ext. ergot in f ʒ ss doses every two hours, was administered as a safe auxiliary to the effects of strychnia. These three cases thus treated all recovered.

The next case coming under my care was a lady seven months advanced in pregnancy, which prevented the use of strichnia or ergot, till premature labor ensued, when it was too late to benefit the patient, as she died within two hours after delivery.

Trusting to the cinchona alkaloids in this malarial disorder, is a measure which greatly hazards the well-doing of the patient, notwithstanding the high authority to the contrary. The following quotations made by Bartholow substantiate this assertion: "Quinine in large doses depresses the heart, arrests it in the diastole without imparing its contractibility, and lower the arterial tension." (Chisom, Briquet.) "Quinine acts on the cardiac motor ganglia, and hence occurs the feebleness of the heart's movements, and in part the general lowering of the vascular tension." (Lewizky.) "Besides these effects, it unquestionably depresses the vaso-motor system, after a short period of preliminary stimulation, probably." (Jerusalimsky, Lewizky, Briquet.) It is clear, then, that the administration of the cinchona alkaloids would increase the vascular expansion already existing. In addition to strichnia being a vaso-motor stimulant, and thereby narrowing the calibre of the blood vessels, it ranks in the third place among the anti-malarial remedies, the first and second places being occupied by quinine and arsenic respectively.

